# Blood Slide Image Analysis to Classify WBC Types for Prediction Haematology Based on a Hybrid Model of CNN and Handcrafted Features

**DOI:** 10.3390/diagnostics13111899

**Published:** 2023-05-29

**Authors:** Fekry Olayah, Ebrahim Mohammed Senan, Ibrahim Abdulrab Ahmed, Bakri Awaji

**Affiliations:** 1 Department of Information System, Faculty Computer Science and information System, Najran University, Najran 66462, Saudi Arabia; 2 Department of Artificial Intelligence, Faculty of Computer Science and Information Technology, Alrazi University, Sana’a, Yemen; 3 Computer Department, Applied College, Najran University, Najran 66462, Saudi Arabia; iaalqubati@nu.edu.sa; 4 Department of Computer Science, Faculty of Computer Science and Information System, Najran University, Najran 66462, Saudi Arabia; balawaji@nu.edu.sa

**Keywords:** deep learning, FFNN, SVM, fusion features, handcrafted features, WBC, haematology

## Abstract

White blood cells (WBCs) are one of the main components of blood produced by the bone marrow. WBCs are part of the immune system that protects the body from infectious diseases and an increase or decrease in the amount of any type that causes a particular disease. Thus, recognizing the WBC types is essential for diagnosing the patient’s health and identifying the disease. Analyzing blood samples to determine the amount and WBC types requires experienced doctors. Artificial intelligence techniques were applied to analyze blood samples and classify their types to help doctors distinguish between types of infectious diseases due to increased or decreased WBC amounts. This study developed strategies for analyzing blood slide images to classify WBC types. The first strategy is to classify WBC types by the SVM-CNN technique. The second strategy for classifying WBC types is by SVM based on hybrid CNN features, which are called VGG19-ResNet101-SVM, ResNet101-MobileNet-SVM, and VGG19-ResNet101-MobileNet-SVM techniques. The third strategy for classifying WBC types by FFNN is based on a hybrid model of CNN and handcrafted features. With MobileNet and handcrafted features, FFNN achieved an AUC of 99.43%, accuracy of 99.80%, precision of 99.75%, specificity of 99.75%, and sensitivity of 99.68%.

## 1. Introduction

Blood cells are one of the basic components of the human body and are divided into WBCs, red blood cells and platelets [1]. Bone marrow produces white blood cells called immune cells [2]. WBCs protect the body from viruses, infections and other infectious diseases. WBCs are an essential component of the immune system and play a crucial role in protecting the body against infections and diseases. There are several types of WBCs, each with distinct functions and characteristics. However, abnormalities in WBC counts or alterations in their morphology can indicate the presence of various diseases. Accurate identification and classification of WBC types are essential for diagnosing specific blood disorders and determining appropriate treatment strategies. WBCs are classified into granular and nongranular cells [3]. Granulocytes (multinuclear) are neutrophils, basophils and eosinophils [4]. Nongranular (mononuclear) cells are lymphocytes and monocytes. An increase or decrease in the number of WBC responses to different diseases depends on the type of WBC [5]. WBC diseases are diagnosed by testing a sample of blood under a microscope. WBCs are broadly classified into several types, including neutrophils, lymphocytes, monocytes, eosinophils and basophils. Neutrophils are the most common type and are involved in fighting bacterial infections. Lymphocytes are responsible for immune responses and can be further categorized as B cells, T cells and natural killer cells. Monocytes play a role in phagocytosis and the immune response to pathogens. Eosinophils are associated with allergic reactions and parasitic infections, while basophils are involved in inflammatory responses and allergies. Abnormalities in WBC counts or alterations in their morphology can indicate various blood disorders such as leukemia, lymphoma, infectious diseases, autoimmune disorder, and allergic reactions. The causes of these diseases can vary, including genetic factors, environmental factors, infections, immune system dysregulation and exposure to toxins. Treatment options for WBC-related diseases depend on the specific diagnosis and may include chemotherapy, radiation therapy, immunotherapy or bone marrow transplantation. Neutrophil cells increase in the blood due to metabolic disturbances, and hormonal conditions. Also, bacteria, fungi, and endogenous and exogenous toxins cause an increase in neutrophil cells in the blood [6]. Hepatitis and whooping cough increase lymphocytes in the blood [7]. Also, HIV, polio, tuberculosis and rubeola decrease lymphocytes in the blood [8]. Monocytes increase in the blood due to diseases such as malaria, listeriosis and viral and bacterial infections [9]. The value of eosinophils in the blood increases due to atopic diseases, allergies and parasites [10]. Conditions associated with basophils include hypothyroidism, malignant myeloproliferative disease, renal disease syndrome and hemolytic anaemia [11].

While lymphocytes, monocytes and neutrophils are involved in various hematological disorders, including some myeloproliferative diseases, it is important to note that the classification of myeloproliferative disorders primarily focuses on abnormalities related to the clonal proliferation of myeloid cells. While lymphocytes, monocytes and neutrophils play essential roles in the immune system and can be affected in various hematological disorders, their involvement in myeloproliferative diseases is not as prominent or defining as in other conditions, such as lymphoproliferative disorders or certain types of leukemia. The specific cell types affected in myeloproliferative disorders are more directly related to the abnormal clonal proliferation of myeloid cells in the bone marrow. Myeloproliferative disorders are a group of hematological conditions characterized by the excessive production of mature myeloid cells in the bone marrow. There are four main types of myeloproliferative disorders recognized by the WHO. Chronic myeloid leukemia (CML) is associated with the presence of the Philadelphia chromosome, resulting in the formation of the BCR-ABL fusion gene. This gene drives the uncontrolled growth and accumulation of mature myeloid cells, particularly granulocytes. Polycythemia vera (PV) is characterized by the overproduction of red blood cells (erythrocytes) in the bone marrow. While neutrophils and other myeloid cells can also be increased, they are not the primary focus of this disorder. Essential thrombocythemia (ET) involves the overproduction of platelets (thrombocytes) in the bone marrow, resulting in increased platelet counts. Myeloid cell abnormalities, including elevated neutrophils and monocytes, can also be observed in some cases, but they are not a defining feature. Primary myelofibrosis (PMF) is characterized by the excessive production of fibrous tissue in the bone marrow, which disrupts normal blood cell production. Neutrophils, monocytes and lymphocytes can be increased, but they are not the primary focus of the disease.

Thus, the diagnosis of many diseases takes place by examining a blood sample for a blood cell count test (hemogram). The hemogram test is based on spreading blood on a glass slide, staining it and then placing it under a microscope to evaluate blood cells [12]. A normal number of WBCs ranges from 4500 to 11,000 units per microliter [13]. In order to identify the types of WBC cells, the hematologist must perform many procedures, such as locating the cells and classifying the type of the microscopic image, which is tedious and time consuming. Also, hematology diagnosis depends on the segmentation and classification of WBC, which is difficult for hematologists because of their irregular sizes and shapes. Thus, potential manual errors and the time it takes to analyze and reach effective results can be reduced thanks to artificial intelligence techniques [14]. The main goal of AI systems is to analyze WBC cells to classify WBC cell types. Based on the analysis of blood samples, automated systems determine the elements of interest for each type of WBC cell and show the morphology of each cell, such as shape, size, colour, texture, and nucleus. Segmentation of WBC cells is a complex task, and the challenge is to select cell boundaries, separate them, and remove artifacts. Many researchers have used machine learning techniques to classify WBC cell types and developed more effective CNN models. 

The use of artificial intelligence (AI) techniques in white blood cell (WBC) classification indeed has significant benefits in the field of pathology. Accurate identification and classification of WBC types are crucial for diagnosing and treating various diseases, including infections, autoimmune disorders, and certain types of cancer. Here are some ways AI enhances WBC classification: AI algorithms analyze large datasets of WBC images and learn to identify and classify different WBC types with high accuracy. This helps pathologists by reducing the chances of human error and providing more consistent and reliable results. AI-powered systems process and classify WBC images much faster than manual methods. This significantly reduces the time required for analysis, allowing pathologists to diagnose and treat patients more efficiently. AI algorithms provide standardized classification criteria, minimizing the subjectivity and variability that may arise when different pathologists interpret WBC images. This leads to more consistent diagnoses and treatment decisions. AI techniques enable the automated screening of large volumes of WBC images, facilitating the identification of rare or abnormal WBCs that may be missed during manual examination. This helps in early detection and diagnosis of diseases. AI systems are used as training tools for pathologists and medical students. By analyzing and comparing large datasets, AI algorithms assist in teaching the subtle differences between different WBC types and improve the skills of practitioners in WBC classification. AI techniques aid in the analysis of vast amounts of data from WBC images, enabling researchers to discover patterns, correlations and new insights into various diseases. This contributes to advancements in pathology and facilitates the development of more targeted treatment strategies. However, it is important to note that AI systems should be used as a supportive tool rather than a replacement for human pathologists. The expertise and clinical judgment of pathologists remains crucial for accurate diagnosis and treatment decisions. AI augments their capabilities and improves the overall efficiency and accuracy of WBC classifications in pathology.

The morphological features of WBC cell types are similar in the early stage, so this study aims to extract hidden features using hybrid techniques between machine and deep learning based on features combined from more than one model.

The most important contributions to this study are as follows:

- Improving blood slide images using overlapping average filters and Contrast limited adaptive histogram equalization (CLAHE);
- Classification of WBC types by SVM based on hybrid features of VGG19-ResNet101, ResNet101-MobileNet and VGG19-ResNet101-MobileNet;
- Classification of WBC types by FFNN based on hybrid features of CNN (VGG19, ResNet101 and MobileNet) and handcrafted features.

The rest of the paper is organized as follows: Section 2 discusses the techniques and findings of previous studies. Section 3 presents the methods and tools for analyzing blood slide images to classify WBC types. Section 4 summarizes the results of WBC type classification systems. Section 5 discusses and compares all strategies for classifying WBC types. Section 6 concludes the study.

## 2. Related Work

Patil et al. [15] employed canonical correlation analysis to classify multiple blood cells. The technique involved the extraction of overlapping nuclei, followed by training and classification using recurrent neural network (RNN) models based on canonical correlation analysis. The authors reported their findings on the successful classification of blood cells using this approach. Yusuf et al. [16] utilized capsule networks for the classification of different types of WBCs. To improve the network’s performance, they fine tuned it and addressed challenges related to overfitting and dataset balancing. The capsule network achieved an impressive accuracy of 96.86% in classifying WBCs. Hüseyin et al. [17] proposed a regional CNN methodology to locate white blood cells and classify them into different types. This approach aimed to facilitate the identification of various white blood cell diseases using the same image. The authors reported the successful implementation of their methodology for WBC classification. Mesut et al. [18] developed a hybrid methodology combining quadratic discriminant analysis and CNN models for WBC classification. Relevant features were extracted using the Ridge test and information coefficient in conjunction with CNN models. The proposed methodology achieved an impressive classification accuracy of 97.95% for WBC types. To address the challenge of limited data, Khaled et al. [19] proposed the use of generative adversarial networks (GANs). This approach aimed to overcome the lack of data, which often hinders system generalization. The CNN models’ weights were initialized either using preselected weights from the CIFAR-100 dataset or randomly. Maxim et al. [20] presented two approaches for classifying WBC species. The first approach involved the classification of hand-extracted features, while the second approach utilized deep learning techniques. The evaluation was performed on spot images using fivefold cross-validation. The reported accuracies for machine learning and deep learning methods were 77.8% and 70.3%, respectively. Ahmed et al. [21] proposed the usage of VGGNet for diagnosing leukemia. The authors highlighted the production of high-level features by VGGNet, which were then filtered using the statistically enhanced Negative Swarm method. The selection of 1000 out of 25,000 features was carried out using the SESS method, resulting in performance improvement. Channabasava et al. [22] introduced the BCNet architecture and fine tuned it using three different optimizers. The performance of BCNet was compared with pretrained CNN models. The results indicated that BCNet with the RMSP optimizer outperformed the other optimizers and pretrained models, demonstrating the superiority of the proposed architecture. Yan et al. [23] developed the WBC-Net network based on the UNet++ architecture and CNN models for WBC classification. WBC-Net incorporated a feature encoder with multiple layers to extract and combine various features using different metrics. The training process involved WBC segmentation improvement through decoding, Tversky index determination, and cross-entropy loss. Partha et al. [24] proposed a method for segmenting the WBC nucleus by employing a colour space transformation and k algorithm. This method facilitated the separation of the nucleus from the rest of the image. The characteristics of the first and last layers of CNN models were collected for WBC type classification. The segmentation method achieved an accuracy of 98.61%, while the CNN model attained 96% accuracy. Asim et al. [25] proposed the utilization of a CNN for the classification of WBCs following image optimization using the CLAHE method. To identify the most discriminative characteristics, the authors employed an optimization technique based on an ant colony, merging the optimized features and subsequently inputting them into an SVM for classification. Cecilia et al. [26] put forth a framework for differential counting of WBCs, aiming to reduce image analysis time and enhance diagnostic efficiency. They employed a colour-shifting technique to highlight WBC cells in the images. The authors performed WBC cell segmentation using the watershed method and extracted chromaticity and texture features. These features were then classified using a random forest classifier. Yusuf et al. [27] introduced a pretrained adaptive model for the classification of WBC species, which leverages knowledge gained from preexisting models. Notably, this adaptive model has the ability to adapt to target domains while disregarding domain differences. Xin et al. [28] proposed a supervised self-learning approach for WBC classification. Their method involved extracting the frontal area of cell images using the K-means clustering method. Subsequently, the WBC area was extracted through concavity analysis. Colour and border features were then extracted using a noncontrast edge optimization operator, and these features were fed into an SVM for classification.

Thus, the researchers devoted their time and effort to achieving superior results for classifying WBC types. This study is distinguished from previous studies by extracting features from several CNN models and integrating them, in addition to incorporating CNN and handcrafted features.

## 3. Materials and Methods 

### 3.1. Description of the WBC Type Dataset

**Figure 1:**
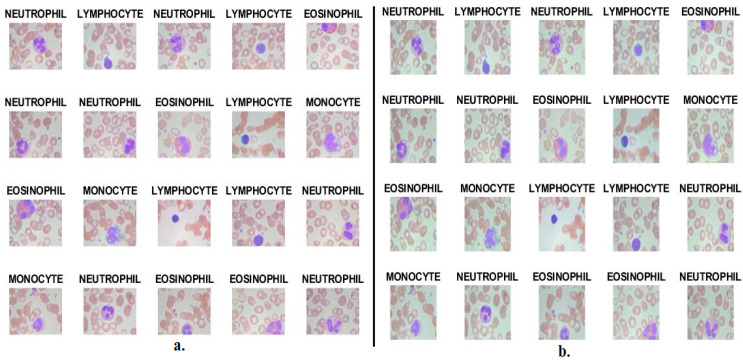
Samples WBC type dataset (**a**) before enhancement, (**b**) after enhancement.

The white blood cell type dataset consisted of 12,507 microscopic images of blood smears on glass slides. The dataset included four types of white blood cells: the Eosinophil type had 3133 images, the Lymphocyte type had 3108 images, the Monocyte type had 3095 images and the Neutrophil type had 3171 images. All images had a 320 × 240 pixels resolution with 24-bit RGB colour space [29]. All images were examined by professional experts and categorized into the four types of WBC. All types of WBC cells contained approximately equal images, so the dataset was balanced. Figure 1a contains random samples from all types of the WBC dataset.

### 3.2. Enhancement Images of Blood Smears for WBC Type

When a blood sample is taken and placed on glass slides under a microscope, noise and artifacts may appear that affect the diagnostic results and cause a breakdown in the performance of CNN models. Mixing the blood sample with eosin and methylene stains is a challenge because of the different colours of the dye. Also, the difference in microscopes, their accuracy and the reflected lights have a negative impact on the performance of the CNN models. Thus, an averaging filter was proposed to remove the noise, and the CLAHE method was presented to show the distorted and blurred edges of the cells.

First, the images of the WBC type dataset were fed into the averaging filter of size 5 × 5. Each time, the filter selected 25 pixels from the WBC image, targeted one pixel called the central, and calculated the average value of the pixels adjacent to the central pixel as in Equation (1). The central pixel was replaced by the average of its neighbors. The filter persisted each time it targeted a central pixel and replaced it with its neighbors [30].

(1) $$f\left(n\right)=\frac{1}{p}\sum_{i=0}^{p-1}s\left(n-i\right)$$

where $f\left(n\right)$ is input, $s\left(n-i\right)$ is the previous input, and *p* is the number of pixels of the average filter.

After that, the image was inserted into the CLAHE method to improve the appearance of the edges. The method brightens the dark edges by distributing the bright regions’ pixels to the dark areas. Each time the central pixel was compared with neighboring pixels, the enhancement was performed as follows: contrast increases when the central pixel was greater than the value of neighboring pixels. At the same time, the contrast decreased when the value of the central pixel was less than that of the adjacent pixels. The method was repeated until all pixels of the image were covered. Thus, an image with prominent edges of WBC cells was obtained. Figure 1b shows images of the blood samples of the WBC dataset after enhancement.

### 3.3. CNN-SVM Technique

This technology introduces a modern mechanism for classifying blood smear images of WBC types using a combination of machine learning and CNN algorithm. The strength of the CNN model lies in the superior capabilities of extracting accurate features that the naked eye cannot detect. Still, one of the challenges of CNN is its need for high-specification devices to train data, which takes a long time. Thus, CNN-SVM technology will solve this challenge [31].

#### 3.3.1. CNN Models for Feature Extraction

The CNN is inspired by multilayer perceptron (MLP) and is designed to learn visual features. They learn features automatically by training the image dataset. Several CNN architectures were created by many layers and their arrangement and steps training, learning rate, and activation functions to classify WBC types. This section describes the CNN architecture, including convolutional layers and the max and average pooling layers. Included are some tunable parameters in the network, the size of the convolutional kernels at each stage [32], the number of tunable filters in each convolutional layer and the size in the average pooling filters. The convolutional layers must have the following: The size of the convolutional filters, several channels as input and output and the convolutional filters equal the input feature map. The convolutional layer applies many filters, learns the weights during the training phase, processes the input and passes it on to the neurons in the next layer. Weights engage neurons that form a convolutional filter. Three parameters that control convolutional layers are the filter size, the zero padding of the input image edges, and the filter step size [33].

Because of the high dimensionality of the features of the convolutional layers, they require complex calculations and a long training time. Thus, pooling layers reduces dimensionality by replacing a group of neurons with a single neuron. There are two types of pooling layers, max pooling and average pooling. The max pooling method selects a group of neurons and replaces it with one neuron that has the maximum value among all neurons as in Equation (2) [34]. The average pooling method selects a group of neurons, computes its average, and replaces the selected neuron with a single neuron, as in Equation (3). Finally, the last layers yield features of sizes 12,507 × 4096, 12,507 × 2048 and 12,507 × 1024 for VGG19, ResNet101 and MobileNet models, respectively. Because of the high dimensions, the best features were selected, and duplicate features were deleted by PCA and saved at sizes of 12,507 × 580, 12,507 × 470 and 12,507 × 410 for VGG19, ResNet101 and MobileNet models, respectively [35].

(2) $$z\left(i;j\right)={max}_{m,n=1\ldots .k}f\left[\left(i-1\right)p+m;\left(j-1\right)p+n\right]$$

(3) $$z\left(i;j\right)=\frac{1}{k^{2}}\sum_{m,n=1\ldots .k}f\left[\left(i-1\right)p+m;\left(j-1\right)p+n\right]$$

where f means size of the filter, m and n are the matrix locations, p means Filter wrap, and k means the vectors.

#### 3.3.2. SVM Algorithm 

**Figure 2:**
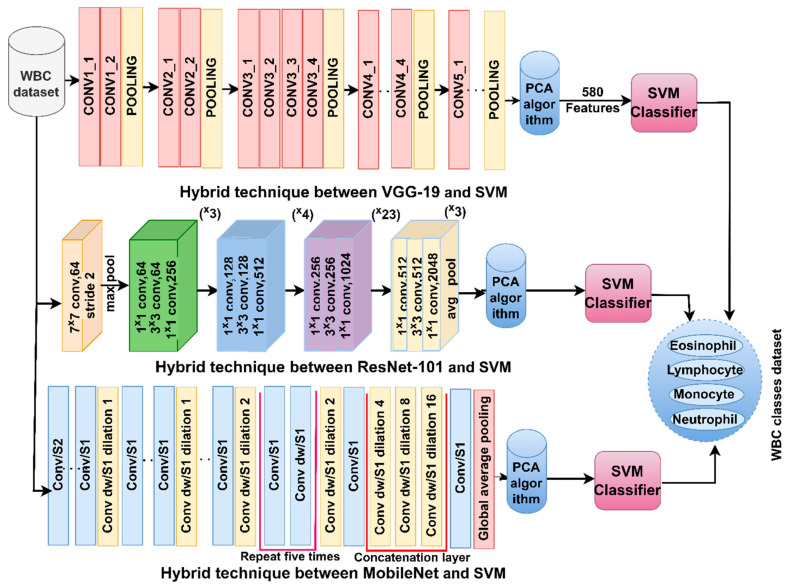
Methodology of blood slides image analysis for classification of a WBC dataset by SVM technique with CNN features.

SVM is based on finding the tradeoff between maximizing the margin and reducing errors in a training set to achieve the best performance classification. SVM aims to create boundaries to separate data into several categories. The best boundaries have the maximum margin between the hyperplane and the data points. SVM works with linearly separable data, which is called linear SVM. Whereas, if the data are not linearly separable, then nonlinear SVM is applied. Support vectors are data points located on decision lines [36]. The SVM algorithm works with binary data by generating one hyperplane to separate the two classes [37]. It also has the ability to work with multiclass data by generating multiple hyperplanes between classes. The SVM takes the features of the VGG19, ResNet101 and MobileNet models, trains them, validates them, and tests their performance. Figure 2 illustrates the methodology for analyzing blood microscopic slide images of the WBC dataset by SVM based on the features of VGG19, ResNet101 and MobileNet.

**Figure 3:**
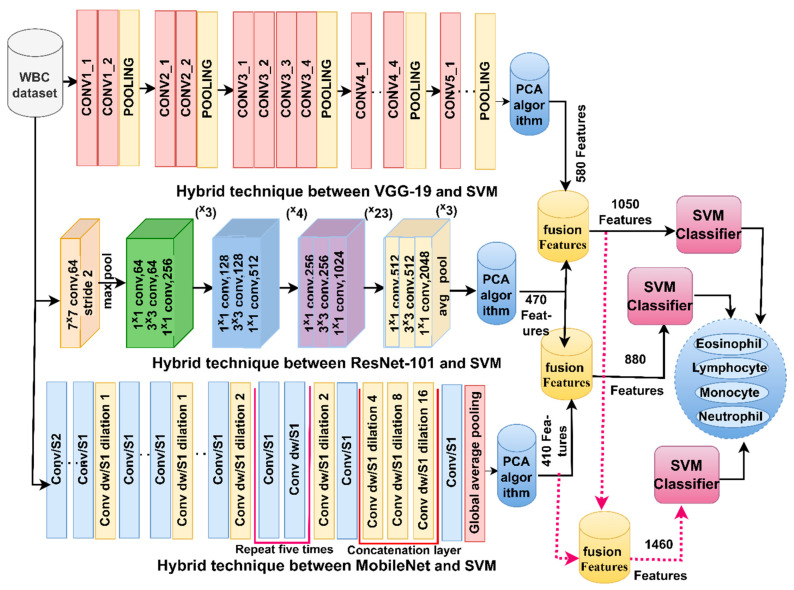
Methodology for analysis of blood slides images for classification of a WBC dataset by SVM technique with features fused to CNN.

Because of the similarity of the biological characteristics of WBC types and for achieving promising results in classifying WBC types, the features of CNN were combined: VGG19-ResNet101, ResNet101-MobileNet and VGG19-ResNet101-MobileNet. It is worth noting that all the features of the CNN models are passed to PCA to select the necessary features. Figure 3 illustrates the methodology for analyzing blood microscopic slide images of the WBC dataset by FFNN based on hybrid features of the CNN models (VGG19-ResNet101, ResNet101-MobileNet and VGG19-ResNet101-MobileNet).

### 3.4. FFNN with Fused CNN and Handcrafted Features

This section presents hybrid systems for classifying WBC types based on hybrid features. The handcrafted features were extracted by hybrid methods GLCM, LBP, DWT and FCH. The study aims to reach superior results for the classification of WBC types, and thus, the features were extracted by CNN, combined with handcrafted features, and then classified by FFNN.

**Figure 4:**
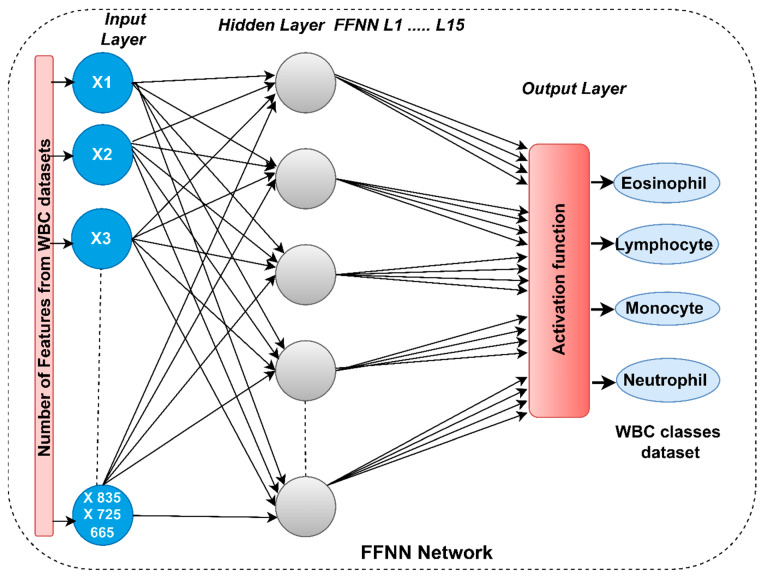
The basic structure of FFNN to classify the WBC dataset.

FFNN is a system consisting of simple, interconnected arithmetic units called neurons. Neurons are interconnected from layer to layer by links, where every connection has weight. At this time, the FFNN has become easy to learn, popular and valuable with complex models like multilayer networks. The FFNN contains an input layer to receive the primary data for a network, 15 hidden layers located between the input and output layer where all the computations are performed, and an output layer to show the results shown in Figure 4. The output value of each neuron is computed through the weights calculated from the neuron’s previous stage. The repetition continues until the minimum square error (MSE) is obtained between the actual and predicted classes, as in Equation (4). The activation is first calculated in nodes in the hidden layers, where each node in the next layer is equal to the sum multiplying the weights with the vectors (nodes) in the previous layer [38].

(4) $$MSC=\frac{1}{n}\sum_{i=1}^{n}\left(x_{i}-y_{i}\right){}^{2}$$

where *n* indicates the number of data points, $x_{i}$ the predicted value and $y_{i}$ is the actual value.

The first step for all systems is to optimize blood slide images for the WBC dataset. The features were extracted from the conventional methods GLCM, LBP, DWT and FCH and combined into a vector as follows:

Extracting texture features from a region of interest according to the GLCM method was provided by Harlick. The GLCM is a method that shows the gray-level composition of WBC cells. Extraction of lesion area texture characteristics by GLCM helps to identify different regions of the WBC cell area. The method distinguishes rough and soft areas based on the value of the central pixel and its neighbors [39]. If the pixels are equal, the cell area is smooth, but if they are different, it is rough. Texture metrics contain spatial information for spatial gray levels, which define the relationship between a pixel and its neighbors based on angle and distance. From each image, 24 features were extracted and saved at a size of 12,507 × 24.

LBP is an efficient texture feature extraction algorithm. LBP selects the target pixel and neighboring pixels, the method specifies the size of 5 × 5 for central pixel analysis, and parameter R specifies the number of adjacent pixels per central pixel. The algorithm compares the central pixel $g_{c}$ with the adjacent pixel $g_{p}$, which are called neighborhood pixels as in Equation (5). Suppose the cell region I (x, y) and $g_{c}$ indicate the gray level of the arbitrary pixel (central pixel), $g_{p}$ indicates the gray level in neighborhoods, R denotes the radius around the central pixel and P is several neighbors [40]. From each image, 203 features were extracted and saved at a size of 12,507 × 203.

(5) $${LBP(x_{c},y_{c})}_{R,P}=\sum_{p=0}^{P-1}s\left(g_{p}-g_{c}\right)\cdot 2^{P}$$

where $g_{c}$ means the center pixel, P means the number of contiguous pixels, $g_{p}$ means the contiguous pixels and R means the contiguous pixels.

Colour is one of the most powerful characteristics that help classify WBC types. The WBC cell colours are distributed over histogram bins, each in one bin. The colours in one container are the same, even if they differ. FCH checks similar colours through the membership value of each pixel and the distribution of pixels across all histogram bins. The fuzzy colour histogram method is used for colour distribution from the point of view of probability [41]. The fuzzy colour histogram method distributes the colour from the probability viewpoint. Consider the colour histogram is represented in the target region I that includes n of pixels as (${X\left(I\right)=x}_{1,}\;x_{2,}\ldots \ldots \ldots x_{i}$) where $x_{i=ni/n}$ which is the probability that the pixels in the image I belong to different colour bins. *ni* indicates the total number of pixels and $i^{th}$ is the total number of colour bins. From each image, 16 features are extracted and saved at a size of 12,507 × 16.

Two-dimensional wavelet transform functions were used to analyze images of WBC types. According to multiple resolutions using quadratic mirror filters, DWT decomposes the input signal into two signals with different frequencies, as in Equations (6) and (7).

(6) $$W_{a,b}=\int_{-\infty }^{\infty }f\left(x\right)\psi_{a,b}\left(t\right)dt$$

(7) $$W_{a,b}(t)=\frac{1}{\sqrt{a}}\psi (\frac{t-b}{a})$$

where *a* and *b* indicate dilating and translating, respectively. 

DWT is obtained when *a* and *b* are specified. The two signals represent wavelet transform functions corresponding to low- and high-pass filters. DWT decomposes the binary signal into four subbands at each level. Each band is passed through a special filter to obtain specific parameters. The first range is passed to the low filter to obtain approximate parameters. The other three subbands pass through high-pass filters (LH, HL and HH) to obtain detailed parameters (horizontal, diagonal, vertical). Through statistical measures of mean, standard deviation and variance, the characteristics of each range are obtained. From each image, 12 features were extracted and saved at a size of 12,507 × 12. 

**Figure 5:**
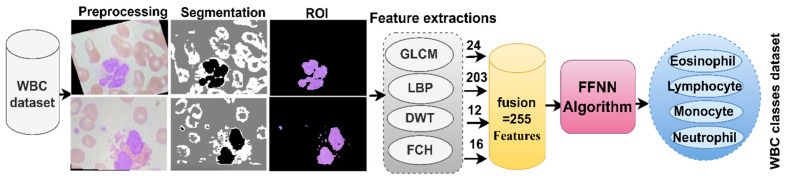
Methodology for analysis of blood slide images for classification of a WBC dataset by the FFNN technique with handcrafted features.

Finally, all method features were combined and saved in vector sizes 12,507 × 255, called handcrafted Features. FFNN received the handcrafted feature vector and distributed it into 80% for network training and 20% to measure network performance. Figure 5 illustrates the methodology for analyzing blood microscopic slide images of the WBC dataset for classifying WBC types by FFNN based on handcrafted features.

**Figure 6:**
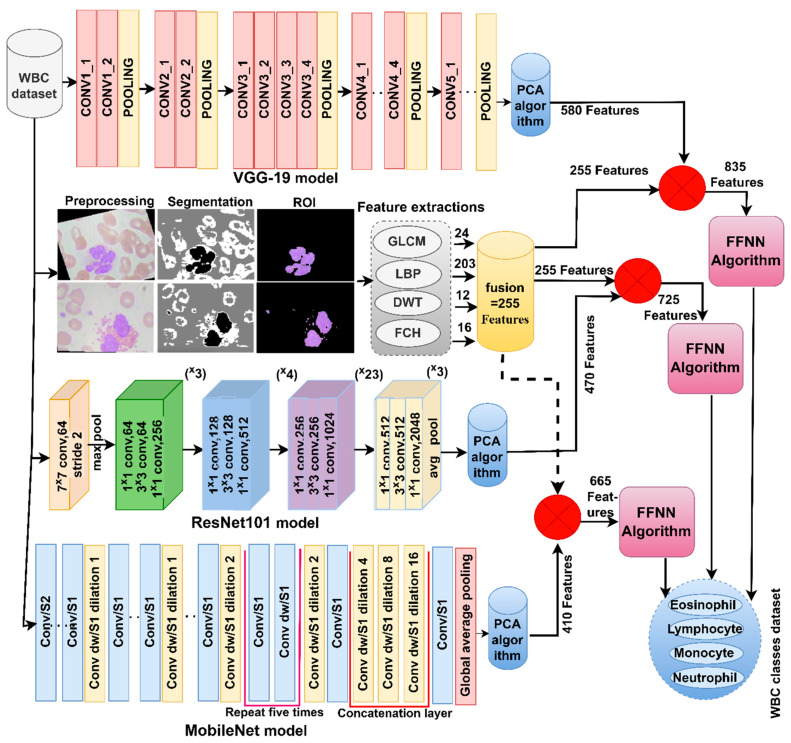
Methodology of blood slide image analysis for classification of a WBC dataset by FFNN technique with features of CNN and handcrafted.

The most important novelty of this study is to achieve superior results for classifying WBC types by integrating features of CNN models with handcrafted features. The first step of this technique was to optimize the images of the WBC dataset and insert them into VGG19, ResNet101 and MobileNet models. The models analyzed blood microscopic images through convolutional layering and pooling described previously. VGG19, ResNet101 and MobileNet models produce high dimensional features; thus, PCA was applied to select the necessary features and eliminate the unnecessary and redundant ones. Finally, features of VGG19, ResNet101 and MobileNet models were obtained and then saved in vectors of sizes 12,507 × 580, 12,507 × 470 and 12,507 × 410, respectively. The VGG19, ResNet101 and MobileNet features were fused separately with the handcrafted features, as shown in Figure 6. Finally, the fused features were introduced to FFNN, which distributed 80% for training the network and validating its generalization, and kept 20% for measuring its performance.

## 4. Results of Techniques Performance

### 4.1. Split of WBC Dataset

All systems were implemented on the WBC dataset. The dataset consists of 12,507 images distributed among four classes of WBC types in a balanced manner. Table 1 shows the WBC type dataset distributed for all systems into 80% for training and validating and 20% for testing the systems.

### 4.2. System Performance Metrics 

The predictive performance of the proposed techniques for classifying the WBC type dataset was measured by evaluative measures described by Equations (8)–(12). The equations show variables that refer to correctly classified samples are called TP and TN, and incorrectly classified samples are called FP and FN [42]. The systems produce a confusion matrix containing all the equations’ variables.

(8) $$AUC=\frac{TPRate}{FPRate}$$

(9) $$Accuracy=\frac{TN+TP}{TN+TP+FN+FP}*100\%$$

(10) $$Precision=\frac{TP}{TP+FP}*100\%$$

(11) $$Specificity=\frac{TN}{TN+FP}*100$$

(12) $$Sensitivity=\frac{TP}{TP+FN}*100\%$$

### 4.3. Results of CNN-SVM Technique

The section discusses the results of CNN-SVM hybrid techniques for analysing blood slide images to classify the WBC typology dataset. This technique depends on two parts: first, the analysis of blood slide images to obtain the morphological characteristics of each type of WBC and extract the hidden features of each image through the convolutional layers of CNN models (VGG19, ResNet101 and MobileNet) and select the essential features using PCA; and second, the essential features of the SVM are then distributed through three phases, 80% for training and validation and 20% from data to test the performance of the SVM.

**Figure 7:**
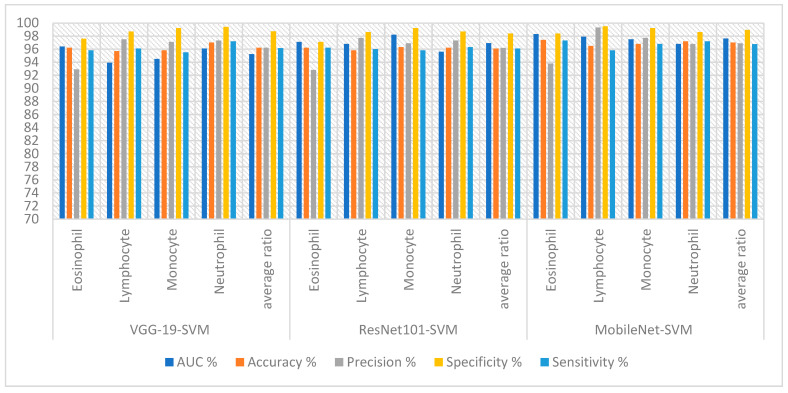
Display performance of SVM-CNN hybrid techniques for analyzing blood slide images to classify a WBC dataset according to the techniques.

The hybrid technique obtained good results for analyzing blood slide images for classifying the WBC type dataset, as shown in Table 2 and Figure 7. SVM yielded the best performance with the features of VGG19. The VGG19-SVM technique achieved an AUC of 95.23%, accuracy of 96.20%, precision of 96.20%, specificity of 98.73% and sensitivity of 96.15%. While ResNet101-SVM achieved an AUC of 96.93%, accuracy of 96.10%, precision of 96.18%, specificity of 98.40% and sensitivity of 96.08%. In contrast, MobileNet-SVM achieved an AUC of 97.63%, accuracy of 97%, precision of 96.90%, specificity of 98.93% and sensitivity of 96.78%.

**Figure 8:**
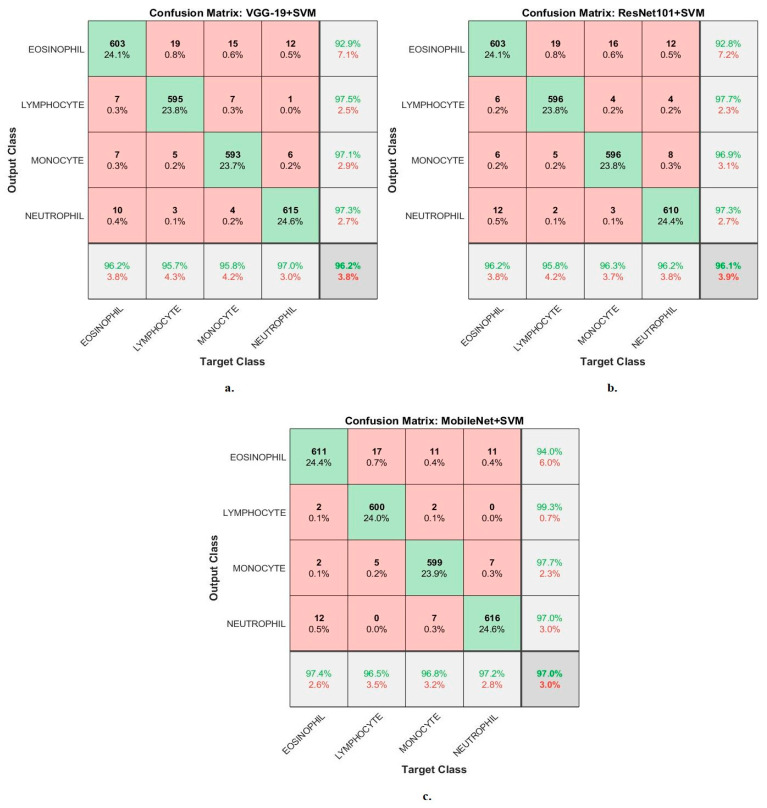
Performance measurement of hybrid techniques for analyzing images of blood slides for classification of the WBC dataset according to the techniques. (**a**) VGG19-SVM; (**b**) ResNet101-SVM; (**c**) MobileNet-SVM.

Figure 8 shows the confusion matrix generated from the VGG19-SVM, ResNet101-SVM and MobileNet-SVM technologies. 

In the confusion matrix, the numbers in the last line represent the accuracy of each class, while the last column represents the precision of each class. The green cells located on the main diagonal represent correctly classified images called TP, whereas the red cells represent the misclassified images called FP and FN.

First, the VGG19-SVM technique achieved accuracy for each WBC type: accuracy for the Eosinophil type of 96.2%, Lymphocyte type of 95.7%, Monocyte type of 95.8% and Neutrophil type of 97%. Second, ResNet101-SVM achieved accuracy for each WBC type: accuracy for the Eosinophil type of 96.2%, Lymphocyte type of 95.8%, Monocyte type of 96.3% and Neutrophil type of 96.2%. Third, the MobileNet-SVM technique achieved accuracy for each WBC type: accuracy for the Eosinophil type of 97.4%, Lymphocyte type of 96.5%, Monocyte type of 96.8% and Neutrophil type of 97.2%.

**Figure 9:**
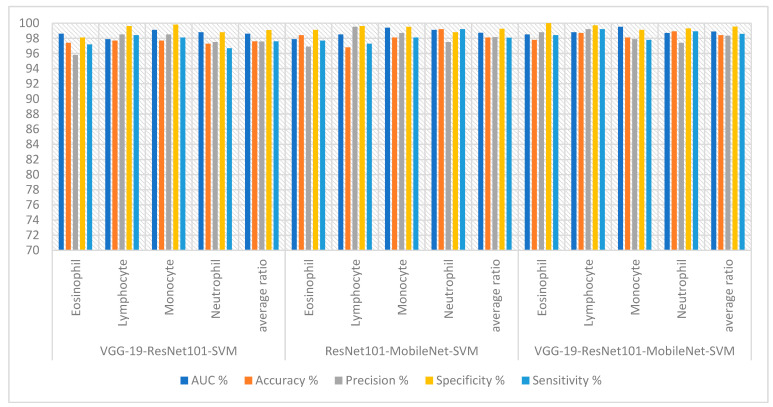
Display performance of SVM with hybrid features of CNN hybrid techniques for analyzing blood slide images to classify a WBC dataset according to the techniques.

The features of CNN models were combined and fed into SVM for classification to achieve a promising accuracy for classifying WBC species. Table 3 and Figure 9 summarize the results for technologies VGG19-ResNet101-SVM, ResNet101-MobileNet-SVM and VGG19-ResNet101-MobileNet-SVM. First, the VGG19-ResNet101-SVM technique obtained an AUC of 98.60%, accuracy of 97.60%, precision of 97.58%, specificity of 99.08% and sensitivity of 97.60%. Second, the ResNet101-MobileNet-SVM technique obtained an AUC of 98.73%, accuracy of 98.10%, precision of 98.15%, specificity of 99.25% and sensitivity of 98.08%. Third, the VGG19-ResNet101-MobileNet-SVM technique obtained an AUC of 98.88%, accuracy of 98.40%, precision of 98.33%, specificity of 99.53% and sensitivity of 98.58%.

**Figure 10:**
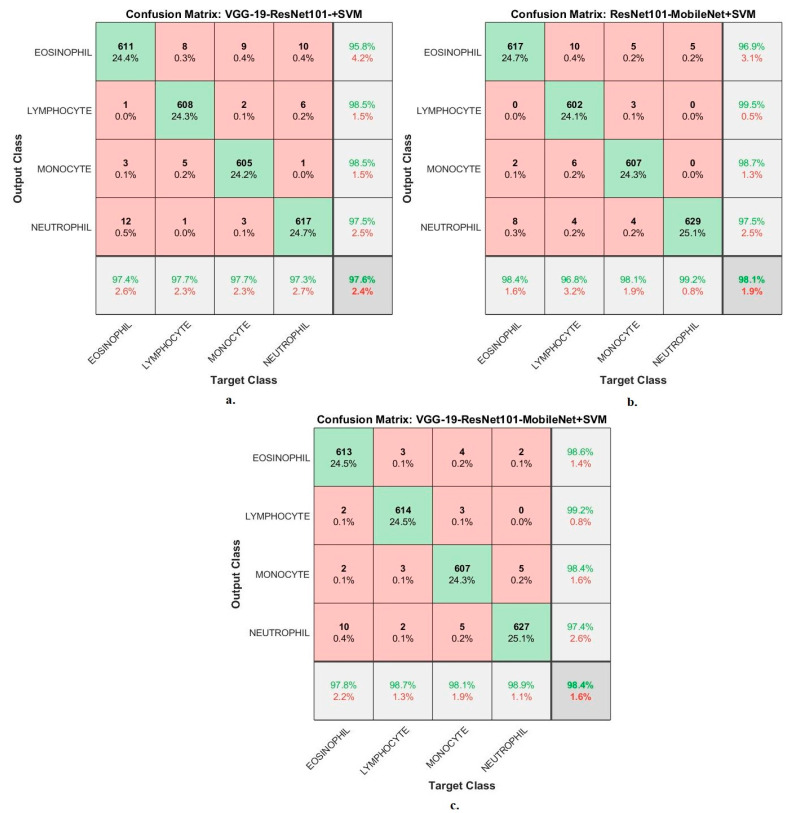
Performance measurement of hybrid techniques for analyzing images of blood slides for classification of the WBC dataset according to the techniques (**a**) VGG19-ResNet101-SVM; (**b**) ResNet101-MobileNet-SVM; (**c**) VGG19-ResNet101-MobileNet-SVM.

Figure 10 shows the confusion matrix generated from the VGG19-ResNet101-SVM, ResNet101-MobileNet-SVM and VGG19-ResNet101-MobileNet-SVM technologies. First, the VGG19-ResNet101-SVM technique achieved accuracy for each WBC type: accuracy for the Eosinophil type of 97.4%, Lymphocyte type of 97.7%, Monocyte type of 97.7% and Neutrophil type of 97.3%. Second, ResNet101-MobileNet-SVM achieved accuracy for each WBC type: accuracy for the Eosinophil type of 97.8%, Lymphocyte type of 98.7%, Monocyte type of 98.1% and Neutrophil type of 98.9%. Third, the VGG19-ResNet101-MobileNet-SVM technique achieved accuracy for each WBC type: accuracy for the Eosinophil type of 97.8%, Lymphocyte type of 98.7%, Monocyte type of 98.1% and Neutrophil type of 98.9%.

### 4.4. Results of FFNN with Fused Features of CNN and Handcrafted

The section discusses the results obtained by the FFNN system with features of CNN and handcrafted features for analyzing blood slide images to classify the WBC classification dataset. This technique is a contribution of the study, which relies on first obtaining the features of the WBC types from the CNN models (VGG19, ResNet101 and MobileNet) and integrating them with the handcrafted features. Second, inputting the essential features of FFNN and then distributing them through three phases: 80% for training and validation and 20% from data for SVM performance testing.

FFNN, based on the handcrafted features, obtained good results for the analysis of blood slide images to classify the WBC type dataset, as shown in Table 4. FFNN yielded an AUC of 93.25%, accuracy of 94.4%, precision of 94.45%, specificity of 98.05% and sensitivity of 94.65%.

**Figure 11:**
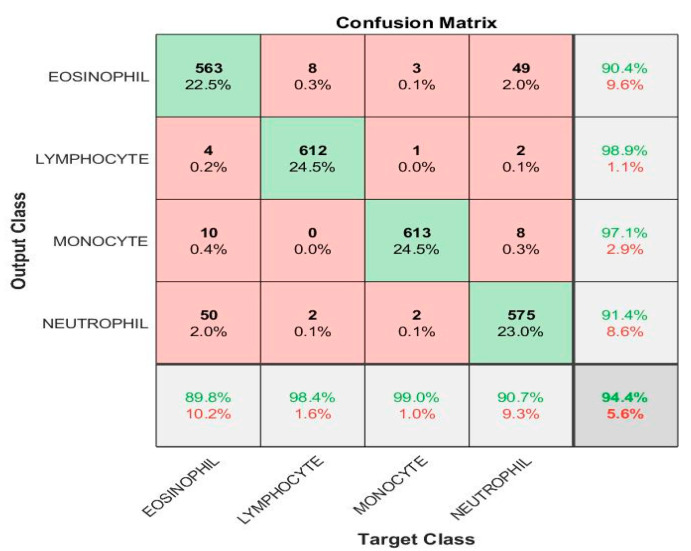
Performance measurement of hybrid techniques for analyzing images of blood slides for classification of the WBC dataset according to FFNN with handcrafted features.

Figure 11 shows the confusion matrix generated by FFNN with handcrafted features. The FFNN technique with handcrafted features achieved accuracy for each white blood cell type: accuracy for eosinophil type 89.8%, lymphocyte type 98.4%, monocyte type 99%, and neutrophil type 90.7%.

**Figure 12:**
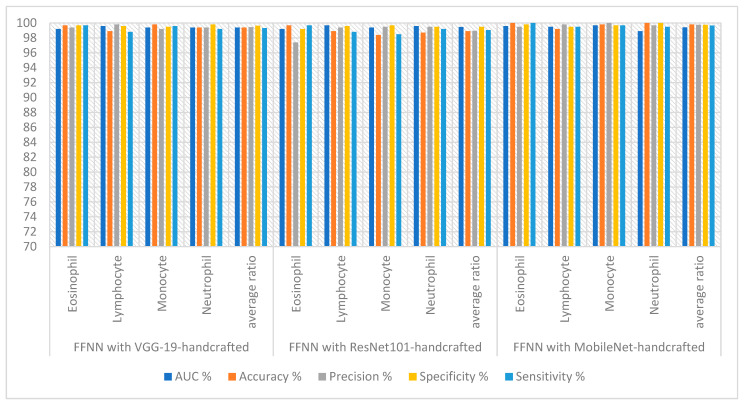
Display performance of FFNN with hybrid features of CNN-handcrafted features for analyzing blood slide images to classify a WBC dataset according to the techniques.

The hybrid technique of FFNN-CNN-handcrafted technique obtained good results for blood slide image analysis to classify the WBC type dataset, as shown in Table 5 and Figure 12. FFNN yielded the best performance with MobileNet and handcrafted features. The FFNN-VGG19-handcrafted technique achieved an AUC of 99.40%, accuracy of 99.40%, precision of 99.45%, specificity of 99.65% and sensitivity of 99.33%. The FFNN-ResNet101-handcrafted technique achieved an AUC of 99.48%, accuracy of 98.90%, precision of 98.95%, specificity of 99.50% and sensitivity of 99.05%. The FFNN-MobileNet-handcrafted technique achieved an AUC of 99.43%, accuracy of 99.80%, precision of 99.75%, specificity of 99.75% and sensitivity of 99.68%.

**Figure 13:**
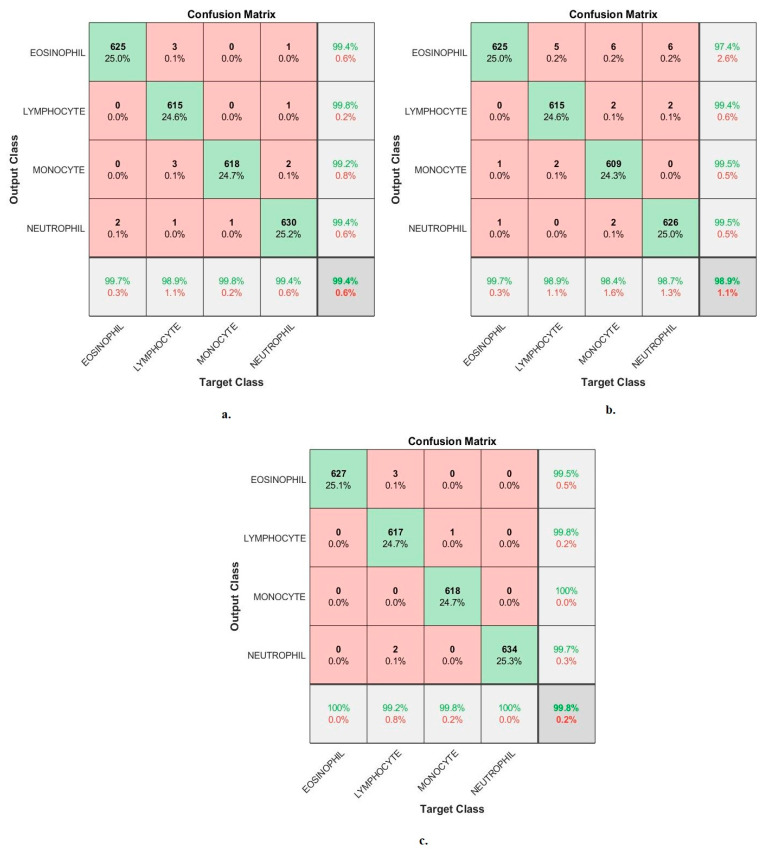
Performance measurement of hybrid techniques for analyzing images of blood slides for classification of the WBC dataset according to FFNN with features of (**a**) VGG19-handcrafted; (**b**) ResNet101-handcrafted; (**c**) MobileNet-handcrafted.

Figure 13 shows the confusion matrix generated by the FFNN-VGG19-handcrafted, FFNN-ResNet101-handcrafted and FFNN-MobileNet-handcrafted technologies. First, the FFNN-VGG19-handcrafted technique achieved accuracy for each WBC type: accuracy for the eosinophil type of 99.7%, lymphocyte type of 98.9%, monocyte type of 99.8% and neutrophil type of 99.4%. Second, the FFNN-ResNet101-handcrafted achieved accuracy for each WBC type: accuracy for eosinophil type of 99.7%, lymphocyte type of 98.9%, monocyte type of 98.4% and neutrophil type of 98.7%. Third, the FFNN-MobileNet-handcrafted technique achieved accuracy for each WBC type: accuracy for eosinophil type of 100%, lymphocyte type of 99.2%, monocyte type of 99.8% and neutrophil type of 100%.

There are also some tools that evaluate FFNN’s performance on the WBC dataset as follows.

#### 4.4.1. Error Histogram

**Figure 14:**
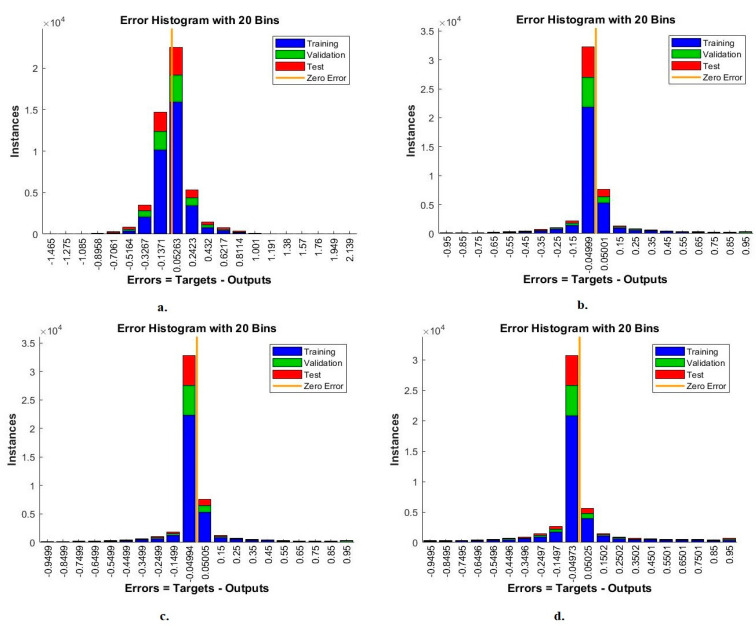
Error histogram for FFNN performance measurement of WBC type dataset classification by FFNN with features for (**a**) handcrafted; (**b**) VGG19-handcrafted; (**c**) ResNet101-handcrafted; (**d**) MobileNet-handcrafted.

The error histogram is one measure that shows the performance of FFNN for analysis of blood slide images for the WBC type dataset. The network records the error between the target and output values in each epoch during the training, testing and validation phases. Each stage appears in a colour distinguished from the other stage, as in Figure 14. The red colour represents the least error during data training, the green colour represents the least error during the data validation stage, and the blue colour represents the least error during data testing [43]. With handcrafted features, the FFNN reached the best performance among 20 bins within the values −1.465 and 2.139. With VGG19 and handcrafted features, FFNN achieved the best performance among the 20 bins with values of −0.95 and 0.95. With ResNet101 and handcrafted features, FFNN achieved the best performance among the 20 bins with the values −0.9499 and 0.95. With MobileNet and handcrafted features, FFNN achieved the best performance among the 20 bins with values of −0.9495 and 0.95.

#### 4.4.2. Cross-Entropy

**Figure 15:**
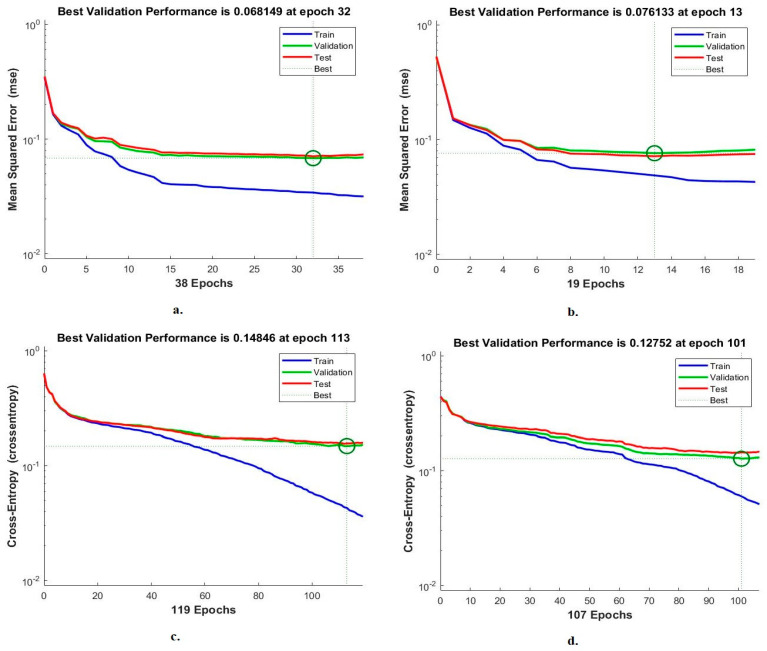
Cross-entropy for FFNN performance measurement of WBC type dataset classification by FFNN with features for (**a**) handcrafted; (**b**) VGG19-handcrafted; (**c**) ResNet101-handcrafted; (**d**) MobileNet-handcrafted.

Cross-entropy is a measure of the performance of FFNN for analysis of blood slide images for the WBC type dataset. The network records the error between the target and output values during each phase. Each stage has a special colour, as in Figure 15. Red represents the least error during data training, green represents the least error during the validation stage, and blue represents the least error during the testing stage [44]. With the handcrafted features, FFNN found the lowest error at epoch 32 with a value of 0.068149. With VGG19 and handcrafted features, FFNN achieved the lowest error at epoch 13 with a value of 0.076133. With the features of ResNet101 and handcrafted FFNN achieved the lowest error at epoch 113 with a value of 0.14846. With MobileNet and handcrafted features, FFNN achieved the lowest error at the epoch of 101 with a value of 0.12752.

#### 4.4.3. Gradient and Validation Checks 

**Figure 16:**
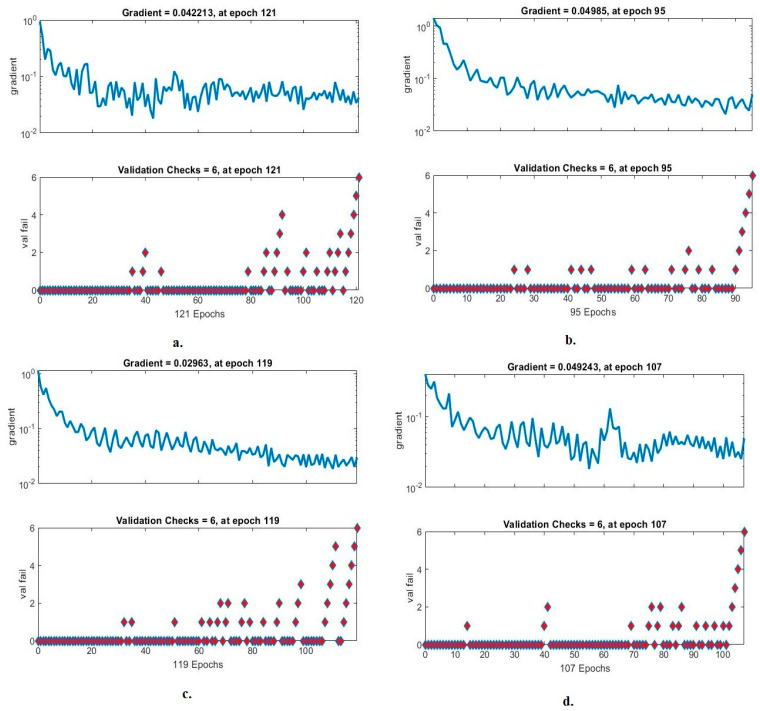
Gradient for FFNN performance measurement of WBC type dataset classification by FFNN with features for (**a**) handcrafted; (**b**) VGG19-handcrafted; (**c**) ResNet101-handcrafted; (**d**) MobileNet-handcrafted.

The validation checks and gradient are metrics that demonstrate the performance of FFNN for analysis of blood slide images for the WBC dataset. In each epoch, the network records the gradient and failures of the FFNN, as in Figure 16. It is noted with the handcrafted features that FFNN achieved a gradient of 0.042213 in epoch 121. With the VGG19 and handcrafted features, the FFNN reached a gradient of 0.04985 in epoch 95. With the ResNet101 and handcrafted features, the FFNN achieved a gradient of 0.02963 in epoch 119. With MobileNet and handcrafted features, the FFNN reached a gradient of 0.049243 in epoch 107.

## 5. Discussion of the Systems Performance for Classifying WBC Types

Recognizing the type of WBC is essential to help pathologists identify the type of disease through blood analysis. Manual analysis requires time, effort and expertise to identify the type of WBC cells. Therefore, AI techniques play a vital role in effective WBC type recognition. In this study, effective hybrid systems were developed to classify blood slide images for the WBC type dataset. The images of all systems were passed to two successive filters to improve the images.

The first technique for analyzing blood slide images is to distinguish between WBC types by CNN-SVM hybrid techniques. WBC images are fed into VGG19, ResNet101 and MobileNet models to extract all accurate features and fed into PCA to select the necessary features. SVM receives the features and classifies them with high accuracy into the four types of WBC. VGG19-SVM, ResNet101-SVM, and MobileNet-SVM techniques reached an accuracy of 96.20%, 96.10% and 97%, respctively, for the classification of the WBC type dataset.

The second technique analyses blood slide images to discriminate between WBC types by hybrid methods of CNN-SVM based on fused features. WBC images are fed into VGG19, ResNet101 and MobileNet models to extract all accurate features and fed into PCA to select the necessary features. Fused features were obtained by combining the features of VGG19-ResNet101, ResNet101-MobileNet and VGG19-ResNet101-MobileNet models. SVM receives fused features and classifies them with high accuracy into the four types of WBC. VGG19-ResNet101-SVM, Res-Net101-MobileNet-SVM and VGG19-ResNet101-MobileNet-SVM techniques reached an accuracy of 97.60%, 98.10% and 98.40%, respectively, for the classification of WBC type dataset.

The third technique is to analyze blood slide images to discriminate between WBC types by hybrid methods based on handcrafted features alone, as well as combining them with CNN models’ features. The WBC images are input into the GLCM, LBP, DWT and FCH methods of extracting the features, merging them, and then inputting them into FFNN for classification achieving an accuracy of 94.4%. The images were analyzed by VGG19, ResNet101 and MobileNet models to extract all the accurate features and input them into PCA to select the necessary features. The VGG19, ResNet101 and MobileNet features are separately integrated with the Handcrafted features. SVM receives melted features and classifies them with high accuracy into the four types of WBC. VGG19-handcrafted-FFNN, ResNet101-handcrafted-FFNN and MobileNet-handcrafted-FFNN techniques reached an accuracy of 99.40%, 98.90% and 99.80%, respectively, for the classification of the WBC type dataset.

**Figure 17:**
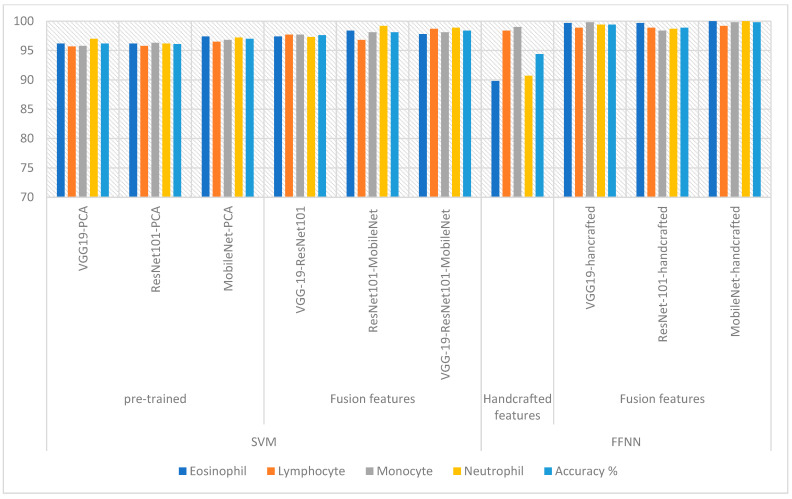
Display of systems performance for classifying the WBC dataset.

Table 6 and Figure 17 summarize the systems performance metrics for classifying the WBC dataset. The systems achieved impressive results in categorizing each type of WBC. FFNN, when fed with the hybrid MobileNet and handcrafted features, achieved the best performance compared to the rest of the systems, which reached an accuracy of 100%, 99.2%, 99.8%, and 100% for the classification of Eosinophil, Lymphocyte, Monocyte and Neutrophil, respectively.

## 6. Conclusions

WBC diseases are a serious health problem. However, several treatments are available that can help improve the quality of life for people with WBC diseases. AI has the potential to improve the diagnosis and treatment of WBC diseases. Deep learning models can be used to extract characteristics from microscopic images of blood smears on glass slides. These characteristics can then be used to determine the type of WBC and distinguish between blood diseases. Artificial intelligence techniques are not a substitute for doctors, but rather help them make accurate diagnoses. Thus, this study dealt with several artificial intelligence systems based on hybrid technologies with hybrid features to classify WBC types. The first technique demonstrates that using individual CNN models combined with SVM can achieve high accuracy in classifying WBC types. The MobileNet model outperformed the other two models with an accuracy of 97%. The second technique improves upon the first technique by combining features from multiple models, resulting in higher accuracy. The fused feature approach using VGG19-ResNet101-MobileNet achieved the highest accuracy of 98.40%. The third technique incorporates both handcrafted features and features from CNN models, resulting in even higher accuracy. By combining the two types of features, the accuracy significantly improves, with the MobileNet-handcrafted-FFNN technique achieving the highest AUC of 99.43%, accuracy of 99.80%, precision of 99.75%, specificity of 99.75% and sensitivity of 99.68%. Overall, the results suggest that using CNN models in combination with SVM or FFNN can effectively classify WBC types in blood slide images. Furthermore, the combination of features from multiple models or the fusion of handcrafted and CNN features can lead to improved accuracy in WBC classification. The highest accuracy achieved was 99.80% using the MobileNet-handcrafted-FFNN technique.

## Figures and Tables

**Table 1:** Splitting the WBC dataset.

Phase	80% (80:20)		Testing 20%
Classes	Training (80%)	Validation (20%)	
Eosinophil	2005	501	627
Lymphocyte	1989	497	622
Monocyte	1981	495	619
Neutrophil	2030	507	634

**Table 2:** Results of the VGG19-SVM, ResNet101-SVM and MobileNet-SVM techniques for classifying the WBC dataset.

Techniques	Classes of WBC	AUC %	Accuracy %	Precision %	Specificity %	Sensitivity %
VGG19-SVM	Eosinophil	96.4	96.2	92.9	97.6	95.8
	Lymphocyte	93.9	95.7	97.5	98.7	96.1
	Monocyte	94.5	95.8	97.1	99.2	95.5
	Neutrophil	96.1	97	97.3	99.4	97.2
	**average ratio**	**95.23**	**96.20**	**96.20**	**98.73**	**96.15**
ResNet101-SVM	Eosinophil	97.1	96.2	92.8	97.1	96.2
	Lymphocyte	96.8	95.8	97.7	98.6	96
	Monocyte	98.2	96.3	96.9	99.2	95.8
	Neutrophil	95.6	96.2	97.3	98.7	96.3
	**average ratio**	**96.93**	**96.10**	**96.18**	**98.40**	**96.08**
MobileNet-SVM	Eosinophil	98.3	97.4	93.8	98.4	97.3
	Lymphocyte	97.9	96.5	99.3	99.5	95.8
	Monocyte	97.5	96.8	97.7	99.2	96.8
	Neutrophil	96.8	97.2	96.8	98.6	97.2
	**average ratio**	**97.63**	**97.00**	**96.90**	**98.93**	**96.78**

**Table 3:** Results of the VGG19-ResNet101-SVM, ResNet101-MobileNet-SVM and VGG19-ResNet101-MobileNet-SVM techniques for classifying the WBC dataset.

Techniques	Classes of WBC	AUC %	Accuracy %	Precision %	Specificity %	Sensitivity %
VGG19-ResNet101-SVM	Eosinophil	98.6	97.4	95.8	98.1	97.2
	Lymphocyte	97.9	97.7	98.5	99.6	98.4
	Monocyte	99.1	97.7	98.5	99.8	98.1
	Neutrophil	98.8	97.3	97.5	98.8	96.7
	**Average ratio**	**98.60**	**97.60**	**97.58**	**99.08**	**97.60**
ResNet101-MobileNet-SVM	Eosinophil	97.9	98.4	96.9	99.1	97.7
	Lymphocyte	98.5	96.8	99.5	99.6	97.3
	Monocyte	99.4	98.1	98.7	99.5	98.1
	Neutrophil	99.1	99.2	97.5	98.8	99.2
	**Average ratio**	**98.73**	**98.10**	**98.15**	**99.25**	**98.08**
VGG19-ResNet101-MobileNet-SVM	Eosinophil	98.5	97.8	98.8	100	98.4
	Lymphocyte	98.8	98.7	99.2	99.7	99.2
	Monocyte	99.5	98.1	97.9	99.1	97.8
	Neutrophil	98.7	98.9	97.4	99.3	98.9
	**Average ratio**	**98.88**	**98.40**	**98.33**	**99.53**	**98.58**

**Table 4:** Results of the FFNN with handcrafted features techniques for classifying the WBC dataset.

Techniques	Classes of WBC	AUC %	Accuracy %	Precision %	Specificity %	Sensitivity %
FFNN-handcrafted features	Eosinophil	92.5	89.8	90.4	96.8	90.4
	Lymphocyte	93.6	98.4	98.9	99.5	98.1
	Monocyte	94.1	99	97.1	98.7	99.3
	Neutrophil	92.8	90.7	91.4	97.2	90.8
	**Average ratio**	**93.25**	**94.40**	**94.45**	**98.05**	**94.65**

**Table 5:** Results of the FFNN with CNN-handcrafted techniques for classifying the WBC dataset.

Techniques	Classes of WBC	AUC %	Accuracy %	Precision %	Specificity %	Sensitivity %
FFNN with VGG19-handcrafted	Eosinophil	99.2	99.7	99.4	99.7	99.7
	Lymphocyte	99.6	98.9	99.8	99.6	98.8
	Monocyte	99.4	99.8	99.2	99.5	99.6
	Neutrophil	99.4	99.4	99.4	99.8	99.2
	**Average ratio**	**99.40**	**99.40**	**99.45**	**99.65**	**99.33**
FFNN with ResNet101-handcrafted	Eosinophil	99.2	99.7	97.4	99.2	99.7
	Lymphocyte	99.7	98.9	99.4	99.6	98.8
	Monocyte	99.4	98.4	99.5	99.7	98.5
	Neutrophil	99.6	98.7	99.5	99.5	99.2
	**Average ratio**	**99.48**	**98.90**	**98.95**	**99.50**	**99.05**
FFNN with MobileNet-handcrafted	Eosinophil	99.6	100	99.5	99.8	100
	Lymphocyte	99.5	99.2	99.8	99.5	99.5
	Monocyte	99.7	99.8	100	99.7	99.7
	Neutrophil	98.9	100	99.7	100	99.8
	**Average ratio**	**99.43**	**99.80**	**99.75**	**99.75**	**99.68**

**Table 6:** Results of systems for analysis of blood slide images for the WBC type dataset.

Techniques	Features		Eosinophil	Lymphocyte	Monocyte	Neutrophil	Accuracy %
SVM	VGG19-PCA		96.2	95.7	95.8	97	96.2
	ResNet101-PCA		96.2	95.8	96.3	96.2	96.1
	MobileNet-PCA		97.4	96.5	96.8	97.2	97
	Fusion features	VGG19-ResNet101	97.4	97.7	97.7	97.3	97.6
		ResNet101-MobileNet	98.4	96.8	98.1	99.2	98.1
		VGG19-ResNet101-MobileNet	97.8	98.7	98.1	98.9	98.4
FFNN	Handcrafted features		89.8	98.4	99	90.7	94.4
	Fusion features	VGG19-hancrafted	99.7	98.9	99.8	99.4	99.4
		ResNet-101-handcrafted	99.7	98.9	98.4	98.7	98.9
		MobileNet-handcrafted	100	99.2	99.8	100	99.8

## Data Availability

Data were collected to support the systems in this study on the WBC type dataset at the following link: https://www.kaggle.com/datasets/alifrahman/main-dataset (accessed on 13 November 2022).

## References

[1] Blood Cells: A Practical Guide-Barbara J. Bain-Google Books. https://books.google.co.in.

[2] Almurayziq T.S., Senan E.M., Mohammed B.A., Al-Mekhlafi Z.G., Alshammari G., Alshammari A., Alturki M., Albaker A. (2023). Deep and Hybrid Learning Techniques for Diagnosing Microscopic Blood Samples for Early Detection of White Blood Cell Diseases. Electronics.

[3] Baghel N., Verma U., Nagwanshi K.K. (2021). WBCs-Net: Type identification of white blood cells using convolutional neural network. Multimed. Tools Appl..

[4] Bosch X., Ramos-Casals M. (2020). Granulocytes: Neutrophils, Basophils, Eosinophils. The Autoimmune Diseases.

[5] Lee Y.-K., Haam J.-H., Cho S.-H., Kim Y.-S. (2022). Cross-Sectional and Time-Dependent Analyses on Inflammatory Markers following Natural Killer Cell Activity. Diagnostics.

[6] Konopleva M.V., Borisova V.N., Sokolova M.V., Semenenko T.A., Suslov A.P. (2022). Recombinant HBsAg of the Wild-Type and the G145R Escape Mutant, included in the New Multivalent Vaccine against Hepatitis B Virus, Dramatically Differ in their Effects on Leukocytes from Healthy Donors In Vitro. Vaccines.

[7] Arend N., Pittner A., Ramoji A., Mondol A.S., Dahms M., Rüger J., Kurzai O., Schie I.W., Bauer M., Popp J. (2020). Detection and differentiation of bacterial and fungal infection of neutrophils from peripheral blood using Raman spectroscopy. Anal. Chem..

[8] Kouli A., Trab S.S., Alshaghel S., Mouti M.B., Hamdoun H. (2020). Congenital nephrotic syndrome as a complication of whooping cough: A case report. Oxf. Med. Case Rep..

[9] Viruses, Plagues, and History: Past, Present, and Future-Michael B. A. Oldstone-Google Books. https://books.google.co.in/books?hl=en&lr=&id=vRP0DwAAQBAJ&oi=fnd&pg=PP1&dq=HIV,+polio,+tuberculosis,+and+rubeola+decrease+lymphocytes+in+the+blood&ots=wQvEHcEi0z&sig=jXsOA9UWhL2PGtj38kDEpuJjzoA#v=onepage&q&f=false.

[10] Théroude C., Reverte M., Heinonen T., Ciarlo E., Schrijver I.T., Antonakos N., Maillard N., Pralong F., Le Roy D., Roger T. (2021). Trained Immunity Confers Prolonged Protection from Listeriosis. Front. Immunol..

[11] Okamoto K., Morio T. (2021). Inborn errors of immunity with eosinophilia. Allergol. Int..

[12] Novakovic T.R., Dolicanin Z.C., Babic G.M., Djordjevic N.Z. (2020). The Maternal Leucocytes in Thrombophilia and Hypothyroidism and their Influence on Fetal Cells. Serb. J. Exp. Clin. Res..

[13] Agarwal R., Sarkar A., Bhowmik A., Mukherjee D., Chakraborty S. (2019). A portable spinning disc for complete blood count (CBC). Biosens. Bioelectron..

[14] Abunadi I., Senan E.M. (2022). Multi-Method Diagnosis of Blood Microscopic Sample for Early Detection of Acute Lymphoblastic Leukemia Based on Deep Learning and Hybrid Techniques. Sensors.

[15] Özyurt F. (2019). A fused CNN model for WBC detection with MRMR feature selection and extreme learning machine. Soft Comput..

[16] Patil A., Patil M., Birajdar G. (2020). White Blood Cells Image Classification Using Deep Learning with Canonical Correlation Analysis. IRBM.

[17] Baydilli Y.Y., Atila Ü. (2020). Classification of white blood cells using capsule networks. Comput. Med. Imaging Graph..

[18] Kutlu H., Avci E., Özyurt F. (2019). White blood cells detection and classification based on regional convolutional neural networks. Med. Hypotheses.

[19] Toğaçar M., Ergen B., Cömert Z. (2020). Classification of white blood cells using deep features obtained from Convolutional Neural Network models based on the combination of feature selection methods. Appl. Soft Comput..

[20] Almezhghwi K., Serte S. (2020). Improved Classification of White Blood Cells with the Generative Adversarial Network and Deep Convolutional Neural Network. Comput. Intell. Neurosci..

[21] Lippeveld M., Knill C., Ladlow E., Fuller A., Michaelis L.J., Saeys Y., Filby A., Peralta D. (2019). Classification of Human White Blood Cells Using Machine Learning for Stain-Free Imaging Flow Cytometry. Cytom. Part A.

[22] Sahlol A.T., Kollmannsberger P., Ewees A.A. (2020). Efficient Classification of White Blood Cell Leukemia with Improved Swarm Optimization of Deep Features. Sci. Rep..

[23] Chola C., Muaad A.Y., Bin Heyat B., Benifa J.V.B., Naji W.R., Hemachandran K., Mahmoud N.F., Samee N.A., Al-Antari M.A., Kadah Y.M. (2022). BCNet: A Deep Learning Computer-Aided Diagnosis Framework for Human Peripheral Blood Cell Identification. Diagnostics.

[24] Lu Y., Qin X., Fan H., Lai T., Li Z. (2021). WBC-Net: A white blood cell segmentation network based on UNet++ and ResNet. Appl. Soft Comput..

[25] Banik P.P., Saha R., Kim K.-D. (2020). An Automatic Nucleus Segmentation and CNN Model based Classification Method of White Blood Cell. Expert Syst. Appl..

[26] Shahzad A., Raza M., Shah J.H., Sharif M., Nayak R.S. (2022). Categorizing white blood cells by utilizing deep features of proposed 4B-AdditionNet-based CNN network with ant colony optimization. Complex Intell. Syst..

[27] Benomar M.L., Chikh A., Descombes X., Benazzouz M. (2021). Multi-feature-based approach for white blood cells segmentation and classification in peripheral blood and bone marrow images. Int. J. Biomed. Eng. Technol..

[28] Baydilli Y.Y., Atila U., Elen A. (2020). Learn from one data set to classify all–A multi-target domain adaptation approach for white blood cell classification. Comput. Methods Programs Biomed..

[29] Zheng X., Wang Y., Wang G., Liu J. (2018). Fast and robust segmentation of white blood cell images by self-supervised learning. Micron.

[30] WBC Multiclass Dataset|Kaggle. https://www.kaggle.com/datasets/alifrahman/main-dataset.

[31] Baig R., Rehman A., Almuhaimeed A., Alzahrani A., Rauf H.T. (2022). Detecting Malignant Leukemia Cells Using Microscopic Blood Smear Images: A Deep Learning Approach. Appl. Sci..

[32] Al-Hejri A.M., Al-Tam R.M., Fazea M., Sable A.H., Lee S., Al-Antari M.A. (2022). ETECADx: Ensemble Self-Attention Transformer Encoder for Breast Cancer Diagnosis Using Full-Field Digital X-ray Breast Images. Diagnostics.

[33] Fati S.M., Senan E.M., ElHakim N. (2022). Deep and Hybrid Learning Technique for Early Detection of Tuberculosis Based on X-ray Images Using Feature Fusion. Appl. Sci..

[34] Lee S.-J., Chen P.-Y., Lin J.-W. (2022). Complete Blood Cell Detection and Counting Based on Deep Neural Networks. Appl. Sci..

[35] Al-Tam R.M., Al-Hejri A.M., Narangale S.M., Samee N.A., Mahmoud N.F., Al-Masni M.A., Al-Antari M.A. (2022). A Hybrid Workflow of Residual Convolutional Transformer Encoder for Breast Cancer Classification Using Digital X-ray Mammograms. Biomedicines.

[36] Senan E.M., Jadhav M.E., Rassem T.H., Aljaloud A.S., Mohammed B.A., Al-Mekhlafi Z.G. (2022). Early Diagnosis of Brain Tumour MRI Images Using Hybrid Techniques between Deep and Machine Learning. Comput. Math. Methods Med..

[37] Maqsood S., Damaševičius R., Maskeliūnas R. (2022). Multi-Modal Brain Tumor Detection Using Deep Neural Network and Multiclass SVM. Medicina.

[38] Senan E.M., Abunadi I., Jadhav M.E., Fati S.M. (2021). Score and Correlation Coefficient-Based Feature Selection for Predicting Heart Failure Diagnosis by Using Machine Learning Algorithms. Comput. Math. Methods Med..

[39] Li B., Xu J., Pan X., Ma L., Zhao Z., Chen R., Liu Q., Wang H. (2022). Marine Oil Spill Detection with X-Band Shipborne Radar Using GLCM, SVM and FCM. Remote Sens..

[40] Zoubir H., Rguig M., El Aroussi M., Chehri A., Saadane R. (2022). Concrete Bridge Crack Image Classification Using Histograms of Oriented Gradients, Uniform Local Binary Patterns, and Kernel Principal Component Analysis. Electronics.

[41] Tian Y., Fang M., Kaneko S. (2022). Absent Color Indexing: Histogram-Based Identification Using Major and Minor Colors. Mathematics.

[42] Senan E.M., E Jadhav M., Kadam A. Classification of PH2 Images for Early Detection of Skin Diseases. Proceedings of the 2021 6th International Conference for Convergence in Technology (I2CT).

[43] Hung C.-C., Lin C.-C., Wu H.-C., Lin C.-W. (2022). A Study on Reversible Data Hiding Technique Based on Three-Dimensional Prediction-Error Histogram Modification and a Multilayer Perceptron. Appl. Sci..

[44] Ahmed I.A., Senan E.M., Shatnawi H.S.A., Alkhraisha Z.M., Al-Azzam M.M.A. (2023). Multi-Techniques for Analyzing X-ray Images for Early Detection and Differentiation of Pneumonia and Tuberculosis Based on Hybrid Features. Diagnostics.

